# Purification of Water Supplied to Towns, Etc.

**Published:** 1856-08

**Authors:** 


*Purification of water supplied to Towns, etc.*—At a recent meeting of the Society of Arts, the method proposed by Dr. Clark for purifying water for the supply of towns was described by him, and its applicability for this purpose discussed.

The substances with which water is contaminated may be in two states—suspended and dissolved; both may contain mineral and organic substance.

Spring water contains from 1-20000 to 1-1000, or even 2-1000, dissolved substance, but no suspended substance. This is the case with many kinds of water in and around London; but, when collected at the surface in reservoirs, and exposed to light and air, vegetation commences, and is succeeded by the development of animalcules. After a time, both the plant and animal organisms pass into a state of putrefaction, and become a source of serious contamination.

The water of rivers generally contains less dissolved substance than that of the springs in the same district, and it also contains suspended substances of various kinds that are washed into the rivers from the banks by small streams, rivers, &c.

The separation of suspended substance is effected either by subsidence or by filtration.

The nature of the dissolved substance depends upon the kinds of strata traversed by the water; it generally consists, for the most part, of calcareous salts—sometimes with magnesian salts—alkaline salts, ammoniacal salts, rarely, and in small amounts.

The calcareous and magnesian salts communicate to water the character of *hardness.* This character varies considerably in amount in different kinds of water, and is expressed in degrees, *each degree of hardness being as much hardness as a grain of chalk, or the lime, or the calcium, in a grain of chalk, would produce in a gallon of water, by whatever meaus it may be dissolved.*

The hardness of most of the water around London is owing to the presence of dissolved carbonate of lime. The amount is so large, that the average supply of water to a single family would yield in eight months 100 pounds of chalk, and in 100 gallons of water there is enough to destroy 35 ounces of soap.

Carbonate of lime itself is very sparingly soluble in water; probably 5000 gallons would be requisite to dissolve one pound avoirdupois. But when combined with an additional amount of carbonic acid, it forms bicarbonate of lime, which is so much more soluble in water, that one pound of carbonate with seven ounces additional of carbonic acid would dissolve in 400 gallons of water; and this is about the amount present in well-water from the chalk strata.

The carbonic acid may be separated from carbonate of lime by heating, as in the ordinary operation of lime-burning, and the lime thus obtained is still more soluble in water than the bicarbonate of lime; so that a pound of carbonate of lime, consisting of—

| | |
|---|---|
| Lime . . . . . . | 9 ounces, |
| Carbonic Acid . . . . . | 7 ounces, |

yields a quantity of lime that may be dissolved in 40 gallons of water.

Thus it appears that carbonate of lime, itself scarcely at all soluble in water, may be rendered soluble in two different ways—either by being deprived of its carbonic acid, or by combining with an additional quantity of carbonic acid.

It is by the latter of these two changes that water, in traversing chalk strata becomes so highly impregnated with carbonate of lime; for carbonic acid is always abstracted from the atmosphere by water during its condensation as rain, &c., and a further amount is frequently dissolved by the water in percolating the vegetable soil.

To separate this dissolved carbonate of lime, so far as may be practicable, is the object of Dr. Clark's method of purification. It is based upon the fact that when a solution of bicarbonate of lime, such as ordinary water, is mixed with a solution of lime, half the carbonic acid is abstracted from the bicarbonate, and both lime and bicarbonate of lime are converted into the very sparingly soluble carbonate.

When this operation is so managed that the lime added is just sufficient to form carbonate with the surplus carbonic acid in the bicarbonate, almost the whole of the dissolved carbonate will be removed from the water, and only so much will remain dissolved as corresponds with the solubility of carbonate of lime.

| | | | |
|---|---|---|---|
| Bicarbonate of lime in 400 gallons | Carbonate of lime 16 oz. | | =2 lbs. |
| | Carbonic acid . . 7 oz. | =16 oz. carb. of lime | |
| Lime in 40 gallons of water | . . . . . . . 9 oz. | | |

This residual carbonate of lime is always small in amount. Supposing in the above instance the 440 gallons contained $1\frac{1}{2}$ oz. dissolved carbonate of lime, 10-11ths, or 16 oz. would be separated, and only $17\frac{1}{2}$ oz. be left in solution. The water, before being softened, would destroy 35 oz. of soap for every 100 gallons; after being softened, the same quantity would destroy only 5 oz.

Most water contains, besides carbonate of lime, calcareous and magnesian sulphates, chlorides, &c. These substances communicate hardness to water, as well as carbonate of lime; but there is this difference—that the hardness, owing to the presence of these substances, is not removed by limeing. This, however, is not of any practical importance, so far as regards the purification of the water supply of London by this method; for the hardness of the water around London is chiefly owing to carbonate of lime.

Without perhaps being prejudicial to health, the disadvantages arising from the presence of carbonate of lime in water, are numerous and considerable.

1. It is the principal cause of the incrustation of steam-engine boilers.

2. It causes a great, and at the same time useless increase in the consumption of soap, and is deposited in dirty linen in such a manner as to fix the dirt, and prevent its being rendered white.

3. For many culinary purposes it is less suitable than soft water.

Dr. Clark's method is remarkable, inasmuch as it differs from most chemical operations in not introducing any other substance into the water in place of the carbonate of lime separated; and moreover, the separation is effected without the use of any substance foreign to the water in its natural state.

There is another effect produced by this method of purifying water, which does not appear to have been at first anticipated by Dr. Clark. It is the removal of organic substance.

In general the wholesomeness of water is much more affected by the presence of organic substance than by mineral substance; and it seems to be a fact well established by observation, that some of the poisons producing epidemic disease find a congenial habitat in water contaminated with organic substance. Moreover, when organic substance in water undergoes putrefaction, the sulphates always present in water are decomposed, and sulphuretted hydrogen generated. The deleterious character of the water of the Niger was ascribed by the late Professor Daniell to this circumstance.

The amount of organic substance in water may be very minute, but it must not on that account be regarded as insignificant. The amount of organic substance in the most defective kinds of water supplied in London, is very small in proportion to the mineral substance; but it is generally considered by recognized authorities, that, under certain conditions, this organic substance may acquire such a state as to produce disease in those drinking it habitually. In this respect the cause of disease existing in water is analogous to that known as sausage-poison, and that producing the frequent fatal effects of a cut with a dissecting knife, neither of which appear to be chemically tangible.

Investigations relating to the last epidemic of cholera have shown that in one district in London, containing a population of 500,000, which were chiefly supplied with water by two different companies, there were over 4000 deaths from cholera during the epidemic. The only recognizable difference in the conditions and modes of life of the inhabitants, was, that one portion were supplied with water of good quality, drawn from a point high up the Thames; while the other portion were supplied with water drawn from a lower point of the river, where it was profusely contaminated with town-drainage. It proved upon inquiry that the mortality among the former portion was 37 in 10,000, while among the other portion it was 130 in 10,000, or three-and-a-half times as great as in those houses supplied with the better water. Further inquiry showed that in the epidemic of 1848–49 the mortality was uniform throughout the district. There was no such difference between the houses supplied with water by the two companies, the mortality being in one case 118 and in the other 125; but at that period both companies drew their water from nearly the same part of the Thames, low down, where it was contaminated with town-drainage.

The method of purification proposed by Dr. Clark not only effects the separation of carbonate of lime, which as regards the wholesomeness of water is of secondary importance, but it also separates organic substance. At the print works, in Manchester, it is applied specially for this purpose, and in an experiment made upon 3,000,000 gallons at the Chelsea Water Works it is stated by Dr. Miller that the amount of organic substance was reduced to one-third.

Some doubt was expressed by speakers who took part in the discussion, as to whether the organic substance removed by limeing was that suspended or that in solution. Both are in fact removed, but it does not appear that there are any grounds for regarding the one more prejudicial to health than the other.

The removal from water of the carbonate of lime dissolved by carbonic acid, has also, indirectly, an influence upon the contamination with organic substance, by serving as a preventive of vegetation, and of the consequent development of animal organism.

When chalk spring water is pumped up from a well and exposed to light and air, in a clean glass vessel, capable of holding a few gallons, with a glass covering, and so exposed that the changes can be observed as they take place from day to day, it will be seen that all around the sides and bottom a green vegetation will appear in summer time within a few days. In process of time this vegetation tends to a brown, and if a close observation be made, a slight incrustation may be discovered, partly to float on the surface of the water, and partly to adhere to the sides and bottom of the vessel. This incrustation consists of carbonate of lime, slowly precipitated from the water by the separation of the duplicate dose of carbonic acid that kept the carbonate of lime dissolved. It is this carbonic acid that serves as the food of plants, furnishing carbon to them, and the carbonate of lime that was kept in solution by it forms the mineral part of the incrustation. If the glass vessel, after having been exposed as described for several weeks, be emptied, a dirty brownish incrustation, including vegetable substance, may be very well seen, all down the sides, and on the bottom. This brownish incrustation has a strong, offensive, marshy smell. If side by side with the spring water there be exposed, in a similar glass vessel, the same water, previously softened, the softened water will continue for weeks and months unaltered, while that unsoftened water is becoming more and more contaminated by vegetation.

So long back as 1851, the commissioners appointed to report on the quality of the water supplied to London, remarked, that "it appeared to be only a question of time, when the sense of the violation of the river purity (by town drainage) would decide the public mind to the entire abandonment of the Thames as a source of supply, unless artificial means of purification were devised and applied." They also stated, "that a careful series of experiments left no doubt in their minds that the means of conducting this process are certain in their results, and sufficiently simple to be left to the execution of a workman of ordinary intelligence, that the process falls easily into the routine operations of water-works . . . is not attended with my peculiar difficulty on the large scale, and that the softening of Thames water in its ordinary condition by this process is perfectly practicable, at a cost which would, on the average, increase the price charged to the consumer only four per cent."

Nevertheless, there is only one instance in which this process has been applied to the purification of water supplied for general purposes. At the Plumstead Water-Works, near Woolwich, it has been in successful operation for the last year and a half. The water supplied by this Company is derived from the chalk by boring, and has about twenty degrees hardness, which is reduced to eight degrees by limeing. The works are capable of supplying 600,000 gallons daily, and at the present time about 3,000 houses are supplied.

Eight months after the Plumstead Water Company had been carrying on the softening process with success, and much to the satisfaction of the consumers, it occurred to the Company to try how far the consumers would continue to be satisfied with the water, if the softening process were omitted.

The consequence was that by the twelfth day the surface of the unsoftened water in the reservoirs, though daily renewed, was covered with masses of confervæ to such an extent, that scarce a square inch could be found clear, and a powerful stench of decaying vegetable substance was evolved. Complaints of the water soon followed, and the experiment was discontinued.

In the course of the discussion Mr. Braithwaite put forward objections to the application of Dr. Clark's method of purification, on the ground that a certain amount of lime was necessary for maintaining the functions of animal life, and cited, in support of his argument, experiments made by Liebig, upon pigeons and cows. But, the quantity of lime supplied in solid food is much more than adequate to these requirements; in many districts, the water consumed by large populations, and by great numbers of cattle, is soft with a very small amount of lime in any state; and further, the lime salt, required for the formation of bone, is not carbonate, but phosphate of lime, which is never present in water to more than an infinitesimal amount. Moreover, the experiments cited by Mr. Braithwaite are quite inapplicable to the case in question, because, in those experiments, lime was entirely abstracted from the solid food, as well as from the water supplied to the animals. —*London Pharmaceutical Journal.*

